# Effect of Goal‐Directed Fluid Therapy on Hypotension From Spinal Anesthesia in Older Parturients Having Cesarean Section: A Randomized Controlled Trial

**DOI:** 10.1155/anrp/2753707

**Published:** 2025-10-28

**Authors:** Jun Ni, Huiying Zhang, Chenyang Xu, Xiali Qian, Huiling Yu, Zijun Tian, Mao Mao, Shanwu Feng

**Affiliations:** ^1^ Department of Anesthesiology, Women’s Hospital of Nanjing Medical University, Nanjing Women and Children’s Healthcare Hospital, No. 123 Tianfei Xiang Mochou Road, Nanjing, 210004, Jiangsu, China; ^2^ Department of Anesthesiology, Gaochun People’s Hospital of Nanjing, Maoshan Road 53 Gaochun District, Nanjing, 211300, Jiangsu, China

**Keywords:** advanced maternal age, cesarean section, goal-directed fluid therapy, hypotension, spinal anesthesia, transthoracic echocardiography

## Abstract

**Background:**

The proportion of advanced maternal age (AMA) parturients in China has gradually increased. AMA is considered a risk factor for adverse maternal and fetal outcomes. Goal‐directed fluid therapy (GDFT) was used to guide perioperative volume management in order to reduce spinal anesthesia‐induced hypotension and optimize maternal and infant outcomes for AMA parturients undergoing cesarean section. The primary endpoint of this study was the incidence of hypotension induced by spinal anesthesia in AMA parturients undergoing cesarean section. Secondary outcomes included intraoperative infusion volume, time to first postoperative flatus, postoperative blood loss within 24 h, neonatal 1‐min and 5‐min Apgar scores, umbilical artery blood gas analysis, and NICU transfer rate.

**Methods:**

A total of 69 AMA parturients with BMI ≤ 35 kg/m^2^ who underwent elective cesarean section with spinal anesthesia were randomly divided into the control group (Group C, *n* = 35) and the GDFT group (Group G, *n* = 34). Group C parturients received compound sodium lactate infusion of 20 mL·kg^−1^·h^−1^ before delivery, which was reduced to 5 mL·kg^−1^·h^−1^ after delivery. Group G parturients were first given compound sodium lactate 3 mL/kg within 3 min after entering the operating room. Thereafter, under the guidance of transthoracic echocardiography (TTE), when the Δ stroke volume (ΔSV) was ≤ 10%, compound sodium lactate was infused at 5 mL·kg^−1^·h^−1^; when the ΔSV was > 10%, the liquid was continued given at 3 mL·kg^−1^·3 min^−1^ until ΔSV ≤ 10%, followed by infusion rate of 5 mL·kg^−1^·h^−1^. The primary endpoint was defined as the incidence of hypotension induced by spinal anesthesia in AMA parturients undergoing cesarean section before anesthesia (T0), after completion of subarachnoid block (T1), at fetal delivery (T2), and at the end of surgery (T3), with hypotension defined as systolic blood pressure (SBP) ≤ 80% of baseline value or mean arterial pressure (MAP) ≤ 65 mmHg. Secondary endpoints included intraoperative infusion volume, time to first postoperative flatus, postoperative blood loss within 24 h, neonatal 1‐min and 5‐min Apgar scores, umbilical artery blood gas analysis, and NICU transfer rate.

**Result:**

Compared with Group C, the amount of predelivery fluid and intraoperative infusion in Group G was significantly reduced (*p* < 0.001), and the incidence of intraoperative hypotension in Group G was significantly decreased (*p* < 0.05). Compared with T0, SBP was significantly decreased at T1–T3 in both groups (*p* < 0.05), SV was significantly decreased at T1 in both groups. Compared with Group C, CO was significantly decreased at T1 in Group G (*p* < 0.05). The first postoperative flatus time was 36.71 ± 10.65 h vs. 31.62 ± 9.19 h, the first ambulation time was 18.06 ± 2.17 h vs. 15.84 ± 2.37 h, and length of stay was 6.37 ± 1.33 days vs. 5.21 ± 1.23 days in Group C and Group G, which were not statistically different, but the first postoperative flatus time of the women in Group G was shortened, which has certain clinical significance. There were no significant differences in neonatal 1‐min and 5‐min Apgar scores, umbilical artery blood gas (other acid–base balance indexes), and NICU transfer rate between the two groups after delivery.

**Conclusion:**

SV‐oriented GDFT based on TTE can reduce the incidence of hypotension after subarachnoid block during cesarean section in AMA parturients. Although there is no significant difference in maternal and infant outcome, the first postoperative flatus time was shortened with a certain degree of clinical significance.

**Trial Registration:**

Chinese Registry of Clinical Trials: ChiCTR2300068420

## 1. Introduction

In recent years, due to policy openness and the improvement of living standards, the proportion of advanced maternal age (AMA) parturients in China has gradually increased. AMA is considered a risk factor for adverse maternal and fetal outcomes. Some AMA maternal comorbidities including gestational diabetes (GDM) and gestational hypertension (HTN) are not rare [1, 2]. The incidence of adverse AMA perinatal outcomes including chromosomal abnormalities, miscarriage, premature birth, admission to the neonatal intensive care unit (NICU), and stillbirth is relatively high [2, 3]. Subarachnoid block is a commonly used anesthesia method during cesarean section, which results in arteriovenous dilation in the innervated area and leads to decreased peripheral vascular resistance, reduced cardiac return blood volume, and decreased cardiac output (CO), thus reducing blood pressure [4]. Fluid load and vasopressors are commonly used in clinical prevention and treatment of hypotension. However, increased plasma volume during pregnancy may aggravate volume load [5, 6], and improper intraoperative fluid management may lead to serious consequences such as pulmonary edema and heart failure [7], especially in AMA parturients with poor compensatory capacity. However, previous research methods and conclusions on liquid load are different, and there is no unified conclusion. Jain et al. [8] showed in a prospective observational study that CO was closely related to uterine placental blood flow, and it is very important to maintain maternal CO during cesarean section. Otherwise, the use of vasopressors may reduce maternal CO, affect uterine placental blood flow, and eventually lead to adverse fetal acid–base imbalance [9]. Although crystalloid loading has a limited effect on preventing hypotension after subarachnoid block, goal‐directed fluid therapy (GDFT) is the most effective form of intravenous fluid therapy [10]. GDFT individualized infusion based on maternal fluid responsiveness to reduce the risk of complications such as pulmonary edema caused by excessive infusion [11]. Controlling stroke volume (SV) change helps to accurately guide intraoperative fluid management [12]. In recent years, TTE has been applied gradually in the perioperative period, which can be used as a measurement tool of hemodynamic indicators during pregnancy [13, 14]. This study aims to reduce spinal anesthesia‐induced hypotension and improve maternal and infant outcomes after cesarean section by GDFT to guide perioperative volume management in AMA parturients.

## 2. General Information

This study was approved by the Medical Ethics Committee of Women ′s Hospital of Nanjing Medical University (Nanjing Women and Children’s Healthcare Hospital) (NO. 2022KY‐128‐01), and all parturients signed an informed consent form. Seventy‐two elective single and full‐term pregnancy cesarean sections under subarachnoid block between February 18, 2023, and March 31, 2023, were selected, with the American Society of Anesthetists (ASA) score of II, BMI ≤ 35 kg/m^2^, and aged 35–45 years.

Exclusion criteria were as follows: (1) cardiac insufficiency, preeclampsia, or uncontrolled hypertension (systolic blood pressure [SBP] ≥ 180 mmHg, DBP ≥ 110 mmHg); (2) preoperative vasoactive drugs are required to maintain vital signs; (3) liver and kidney insufficiency; (4) unable to obtain clear and qualified TTE images; (5) failure of spinal block puncture or dissatisfied anesthetic effect; (6) parturients request to terminate the trial; (7) massive bleeding with bleeding volume > 800 mL occurred during the operation.

## 3. Study Design

### 3.1. Anesthetic Protocol

After entering the operating room (OR), the upper limb vein was punctured with 18‐G indenting needle to establish venous access. SBP, DBP, mean arterial pressure (MAP), HR, SV, CO, and SpO_2_ were monitored. If SpO_2_ < 95%, oxygen inhalation was given by the mask. A 16‐G needle was used first to locate the epidural cavity, then a 25‐G lumbar puncture needle was used to puncture through the inner lumen of the epidural needle into the subarachnoid space.     Once cerebrospinal fluid outflow was noted, a mixture composed of 1 ml of 10% hypertonic glucose solution (GS) and 2 ml (15 mg) of 0.75% ropivacaine (Nelapine®, AstraZeneca) was injected into the subarachnoid space. The injection speed was set at 10 seconds per milliliter.[15]. After completing the local anesthetics, the parturient was returned to a left‐leaning 15° supine position and inhaled oxygen through a nasal cannula at 2 L/min. The temperature sensory block plane was tested with an alcohol swab, and the operation was started when the block plane reached the T4 level or above. If the block plane was insufficient within 10 min, the parturient was excluded from the study. When the HR is below 50 times per minute, 0.5 mg of atropine should be given intravenously. If the parturient developed hypotension after subarachnoid block (SBP ≤ 80% of the baseline value) and HR ≥ 60 times/min, the parturient was given phenylephrine, 0.1 mg each time, and repeated as necessary until SBP rose to more than 80% of the baseline value.

The amount of blood loss during cesarean section was estimated by the gauze weighing method combined with the standard attractor quantitative method. The gauze to be used in the operation was weighed and recorded with a fixed electronic scale preoperatively, and the gauze soaked with blood was weighed postoperatively, and the subtractive difference was calculated as 1 g (blood weight) = 1 mL (blood volume). In addition, the total amount of blood in the standard aspirator was recorded, while amniotic fluid was collected in another separate suction device after rupture of the membrane. When gauze weighing and standard attractor quantities were added together, the amount of bleeding was estimated. After delivery, routine intramuscular injection of oxytocin 10 U, intravenous infusion of oxytocin 10 U, and depending on the uterine contraction situation decide whether to add the uterine contraction‐promoting drugs such as carbetocin or carboprost amine triol injection.

The personnel involved in implementing the interventions in this study underwent rigorous selection and specialized training. Anesthesiologists, responsible for executing the fluid therapy protocols, each possessed over 5 years of clinical anesthesia experience and had completed basic training in TTE along with specialized training in GDFT.

Prior to the study, all personnel passed simulated operational assessments to ensure proficiency in TTE monitoring techniques and criteria for assessing fluid responsiveness. Additionally, the research team held regular meetings to review operational details, ensuring consistency and accuracy in intervention delivery.

### 3.2. Grouping and GDFT

**Figure 1:**
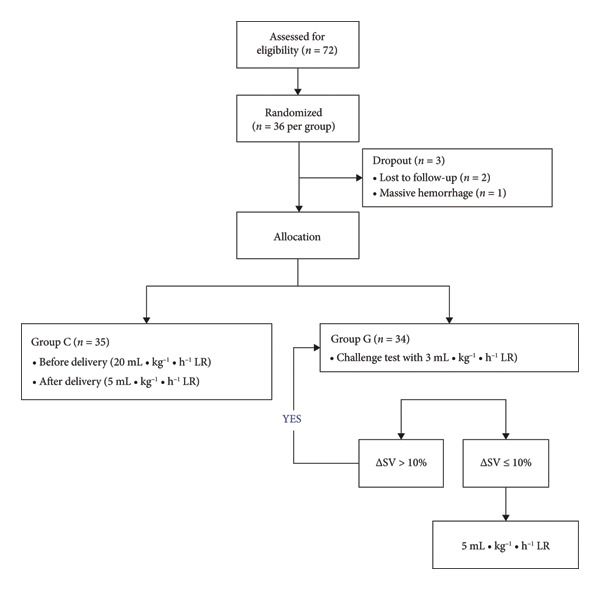
Flow chart.

SV represents the volume of blood ejected from the left ventricle with each contraction. ΔSV is calculated as the percentage change in SV from a baseline measurement. Specifically, it is determined by the formula: ΔSV = (SVpost‐SVpre)/SVpre × 100%, where SVpre is the SV measured at the baseline time point and SVpost is the SV measured after a certain intervention or time interval. The selection of a 10% threshold for ΔSV as the intervention criterion in this study is based on previous research. According to the study by [Author’s name et al., Year, Reference number], a 10% change in SV has been shown to be a clinically significant indicator in GDFT. This threshold can effectively distinguish between patients who require fluid resuscitation and those who are adequately volume‐loaded, thereby optimizing fluid administration and improving patient outcomes [13, 14]. Seventy‐two parturients with BMI ≤ 35 kg/m^2^ who underwent elective cesarean section were randomly divided into the control group (Group C) and the GDFT group (Group G). The envelope randomization method was adopted, a random number was first generated and marked on the envelope, and the appearance of the envelope will not differ. After parturients are enrolled, the envelopes are opened in order and the parturients are assigned to Group C or Group G based on the numbers inside, which ensures randomization and avoids human interference. Group C received compound sodium lactate (China Otsuka Pharmaceutical Co., Ltd., 22K73F5) of 20 mL·kg^−1^·h^−1^ before delivery, which was reduced to 5 mL·kg^−1^·h^−1^ after delivery. Group G was given compound sodium lactate 3 mL/kg within 3 min (challenge test) [16] after entering the OR, under the guidance of TTE (Philips Ultrasound Inc., WA 98021, US). When the change in stroke volume (ΔSV) is ≤ 10%, infuse compound sodium lactate at a rate of 5 ml·kg⁻¹·h⁻¹. When the ΔSV is > 10%, indicating a positive fluid response, continue to infuse lactate at a rate of 3 ml·kg⁻¹·3 min⁻¹ until the ΔSV ≤ 10%. Then, adjust the infusion rate to 5 ml·kg⁻¹·h⁻¹.Clinicians performing TTE are not responsible for fluid infusions and the use of vasopressors. Initially, a cohort of 72 parturients was enrolled in this study. Following randomization, each group comprised 36 parturients. Throughout the study duration, one participant from each group failed to maintain follow‐up. Furthermore, one participant from Group G was excluded owing to significant intraoperative bleeding triggered by adhesions stemming from prior multiple abdominal surgeries. Consequently, a total of three participants were excluded from the trial. Ultimately, the analysis incorporated 35 cases from Group G and 34 cases from the other group, with successful follow‐up achieved for the remaining 69 cases (Figure 1).

## 4. Observation Items and Indicators

Intraoperative blood loss, urine volume, infusion volume, intraoperative hypotension, nausea, and vomiting were recorded. SBP, DBP, MAP, HR, SV, and CO were recorded at the time of entry (T0), after subarachnoid block (T1), fetal delivery (T2), and at the end of surgery (T3). Apgar score of newborns 1 and 5 min was recorded. Results of umbilical artery blood gas analysis (including pH, PaCO2, PaO2, BE, and Lac) and neonatal transfer to NICU were recorded. The time of first postoperative flatus, the time of first postoperative ambulation, length of stay, and the amount of postoperative blood loss and urine volume within 24 h were also recorded. The first postoperative flatus time refers to the time when the patient’s intestinal function begins to recover and the gas accumulated in the intestine is expelled for the first time after undergoing surgery. In this study, hypotension was defined as a SBP ≤ 80% of the baseline value or a MAP ≤ 65 mmHg. ΔSV variation was monitored via TTE and calculated using the formula: ΔSV = (Current SV‐Baseline SV)/Baseline SV × 100%. When ΔSV ≤ 10%, it indicated a negative fluid responsiveness, prompting a reduction in infusion rate; when ΔSV > 10%, it indicated a positive fluid responsiveness, prompting an increase in infusion rate until ΔSV ≤ 10%. These definitions and calculations ensured precision in hypotension assessment and fluid management.

This study included a postoperative follow‐up period of 7 days, conducted through outpatient reviews, telephone interviews, and medical record reviews. The follow‐up covered maternal recovery, complications, and neonatal health status to ensure a comprehensive assessment of postoperative outcomes and robustness of the data collected.

## 5. Statistical Analyses

According to the results of the previous study, the nonhypotensive rate in the conventional infusion group was 34%, the nonhypotensive rate in the GDFT group was 66%, and the set *α* = 0.05, *β* = 0.1, and the shedding rate *λ* = 0.2 were set. The total sample size was 72 cases, 36 cases in Group C and 36 cases in Group G.

According to the intention‐to‐treat (ITT) principle, which states “once randomized, always analyzed,” all 72 patients should have been included in the analysis. However, due to the specific circumstances in this study, we adopted the modified intention‐to‐treat (mITT) principle. The mITT principle permits the exclusion of patients who did not receive the study intervention. In our study, during the study period, 1 case in each group was lost to follow‐up. Additionally, 1 case in Group G was excluded due to intraoperative massive hemorrhage caused by adhesions resulting from multiple abdominal operations. These three patients did not fully receive the study‐related interventions as planned. Therefore, to ensure the accuracy and reliability of the analysis results, we excluded these noncompliant cases based on the mITT principle. Finally, 35 cases in Group G and 34 cases in the other group were included in the analysis and follow‐up of the remaining 69 cases was completed successfully. We believe that this approach adheres to scientific rigor and provides a more accurate reflection of the treatment effects under actual study conditions.

SPSS 25.0 statistical software was used for data processing. Normal distribution measurement data were expressed as mean ± standard deviation ($\overset{¯}{x}$ ± *s*), independent sample *T* test was used for comparison between groups, and repeated measurement analysis of variance was used within groups. Non‐normal distribution measurement data were expressed as median (M) and interquartile distance (IQR), and Mann–Whitney *U* test was used for comparison between groups. Qualitative data were presented as counts (*n*) and percentages (%), and the chi‐square test or Fisher’s exact probability method was used for comparison between groups, when *p* < 0.05 was considered statistically significant.

Adjustments for multiple comparisons were not made, considering the number of endpoints, which should be acknowledged as a limitation. Confidence intervals for key results have been included to support the *p* values, enhancing the robustness of the conclusions.

## 6. Results

A total of 72 parturients were included in this study at the beginning. After randomization, there were 36 parturients in each group. During the study, 1 case in each group was lost to follow‐up. In addition, 1 case in Group G was excluded due to intraoperative massive hemorrhage caused by adhesions resulting from multiple abdominal operations. In total, 3 cases were excluded from this trial. Finally, 35 cases in Group G and 34 cases in the other group were included in the analysis, and follow‐up of the remaining 69 cases was completed successfully. All enrolled parturients met the indications for cesarean section and did not receive drugs that promote uterine contraction, such as carboprost and triol injection. Surgical time ranged from 0.5 to 1.5 h, with no difference between the two groups. There was no difference between the two groups in the amount of phenylephrine. The subarachnoid anesthesia level was between T5 and T8, and there was no difference between the two groups.

### 6.1. General Data

1. There was no significant difference in maternal age, BMI, pregnancy, preoperative fasting time, and other general data between the two groups. There was no significant difference in 24‐h blood loss, 24‐h urine volume, and other postoperative general data between the two groups (Table 1).
2. Compared with Group C, the amount of predelivery fluid and intraoperative infusion in Group G was significantly reduced, and the incidence of intraoperative hypotension was significantly decreased (*p* < 0.05). (Table 1).

### 6.2. Vital Signs

1. Compared with Group C, the HR was significantly increased at T2 (*p* < 0.05) and CO was significantly decreased at T1 in Group G (*p* < 0.05), respectively. There were no significant differences in BP, SV, and CO in both groups and there was no significant difference at other time point (Figure 1, a: SBP, b: DBP, c: MAP, d: HR, e: SV, f: CO.).
2. Compared with T0, SBP was significantly decreased at T1‐T3 in both groups (*p* < 0.05), and SV was significantly decreased at T1 in both groups (Figure 2).

### 6.3. Related Indicators of Maternal and Infant Outcomes

1. The first postoperative flatus time was 36.71 ± 10.65 h vs. 31.62 ± 9.19 h, the first ambulation time was 18.06 ± 2.17 h vs. 15.84 ± 2.37 h, and length of stay was 6.37 ± 1.33 days vs. 5.21 ± 1.23 days for Group C and Group G, which was not statistically significant (Figure 3). (*p* > 0.05).
2. Adverse reactions such as dizziness, nausea, vomiting, and chest tightness occurred occasionally; however, there were no significant differences between two groups. Pulmonary edema did not occur in the two groups. There were no significant differences in neonatal Apgar score at 1 min and 5 min, and NICU transfer rate between the two groups after delivery (Table 2).
3. There were no significant differences in neonatal umbilical artery blood gas (other acid–base balance indexes) (Figure 4).

## 7. Discussion

AMA refers to those who undergo delivery at 35 years of age and above. This delay in childbirth is believed to be caused by social changes, such as the implementation of oral contraceptives, uterine surgeries, prioritizing education and career development for women of childbearing age, and accessing assisted reproductive service technologies [17]. A study [18] found that the AMA incidence rates of gestational HTN, GDM, placenta previa, and eclampsia were 7.8%, 28.48%, 2.2%, and 4.6%, respectively, which were all higher than those of the appropriate age group. At the same time, a retrospective study of age and risk of cesarean sections in Northern Europe [19] found that the rate of cesarean section increased from 14.0% for first‐time parturients aged 20–39 years to 39.9% for parturients aged 40 years and older, and 3.9% for parturients aged 20–39 years and 9.1% for parturients aged 40 years and older. The proportion of cesarean section in AMA parturients was significantly higher than that in the appropriate age group. Therefore, the rate of complications during pregnancy and postpartum complications has increased significantly, so it is necessary to pay attention to the perinatal safety of AMA parturients. AMA are often prone to hypotension due to physiological variability and the vasodilation effect of spinal anesthesia, which not only threatens the safety of the parturients but may also affect the health of the fetus.

**Table 1:** Comparison of general data of parturients in two groups.

	Group C (*n* = 35)	Group G (*n* = 34)	*p* value
Age	36.77 ± 1.88	37.15 ± 1.84	0.41
BMI	26.62 ± 2.85	26.54 ± 2.63	0.90
Gestational age (day)	271.23 ± 6.95	271.29 ± 5.93	0.98
Preoperative fasting time (hours)	9.94 ± 2.12	9.78 ± 2.41	0.77
Preoperative and postoperative fasting time (hours)	16.06 ± 2.17	15.84 ± 2.37	0.69
The amount of predelivery fluid (mL)	350 (300–400)	285 (247–302)	< 0.001
Intraoperative fluid (mL)	572.57 ± 105.48	475.88 ± 83.20	< 0.001
Intraoperative urine (mL)	154.71 ± 40.82	134.12 ± 32.67	0.024
The amount of bleeding (mL)	444.00 ± 83.39	423.82 ± 59.85	0.25
24 h blood loss (mL)	599.94 ± 210.52	610.74 ± 139.94	0.80
24 h urine volume(ml)	2302.86 ± 447.86	2510.29 ± 615.31	0.11
Incidence of hypotension (%)	16 (45.7%)	7 (20.6%)	0.04

Figure 2Comparison of BP, SV, and CO values between two groups. ^∗^: *p* < 0.05 (comparison between groups); ^#^: *p* < 0.05 (compared to T0); ^&^: *p* < 0.05 (between different time points within the same group).

**Figure:**
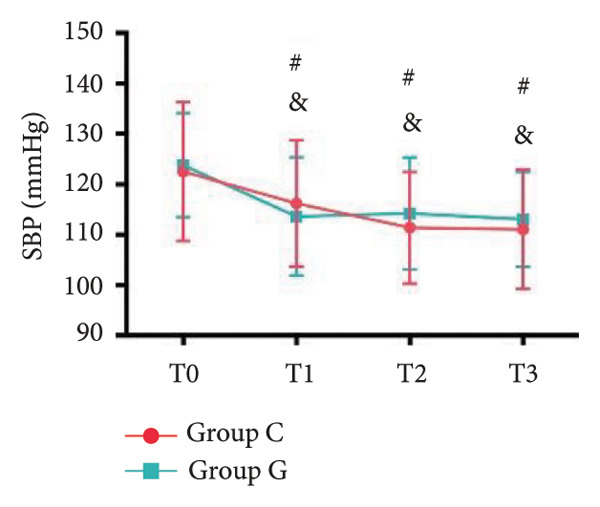
(a)

**Figure:**
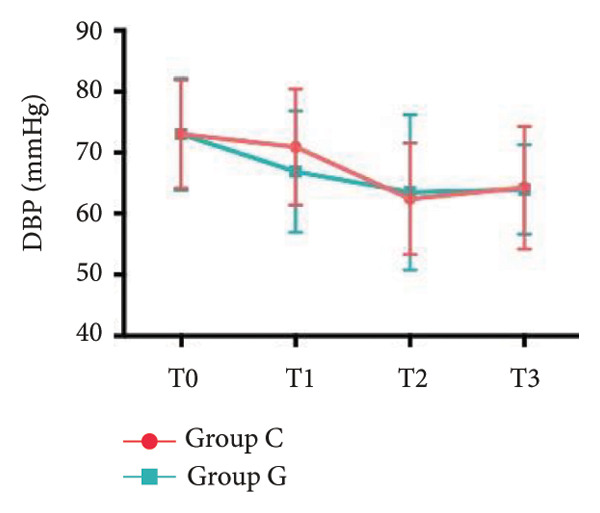
(b)

**Figure:**
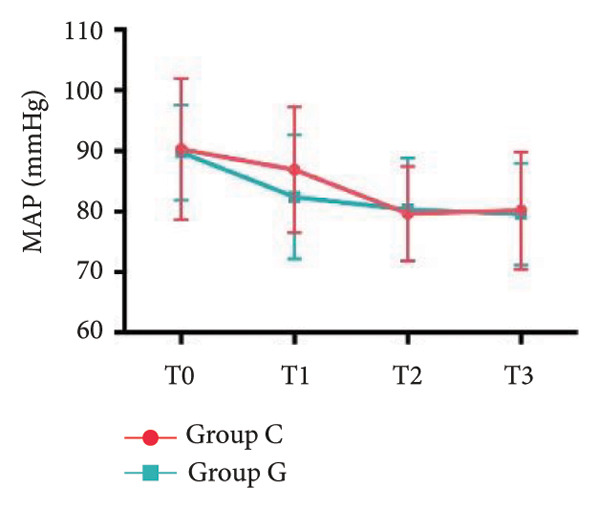
(c)

**Figure:**
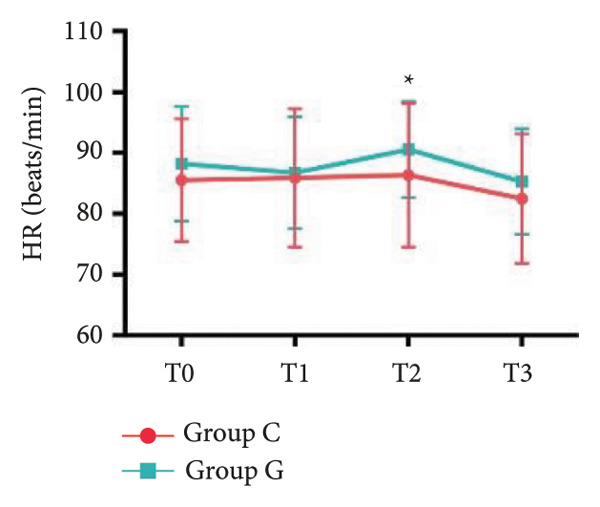
(d)

**Figure:**
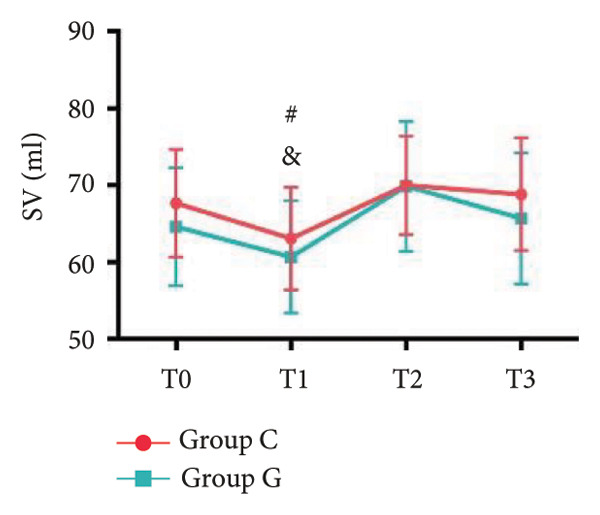
(e)

**Figure:**
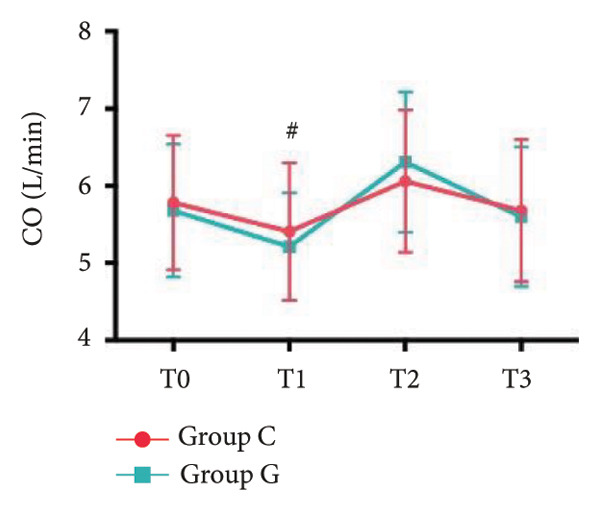
(f)

Figure 3Comparison of postoperative first flatus time, first ambulation, and length of stay between two groups.

**Figure:**
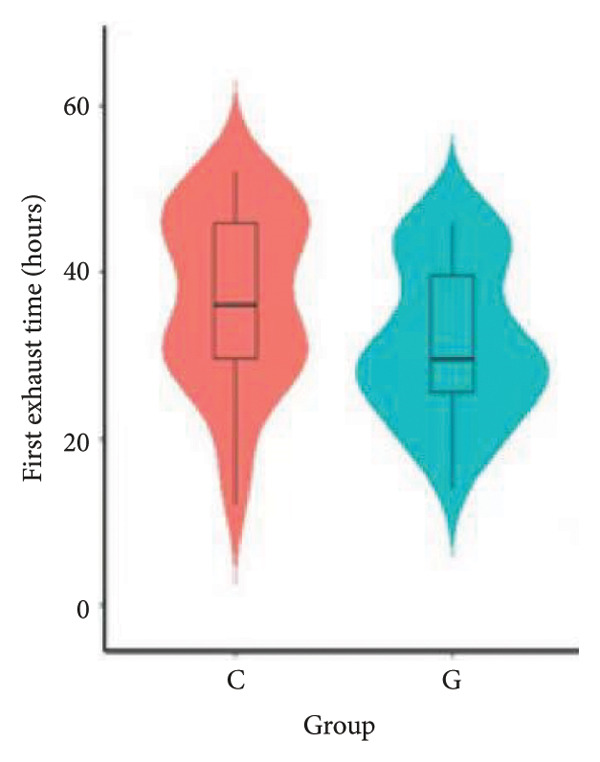
(a)

**Figure:**
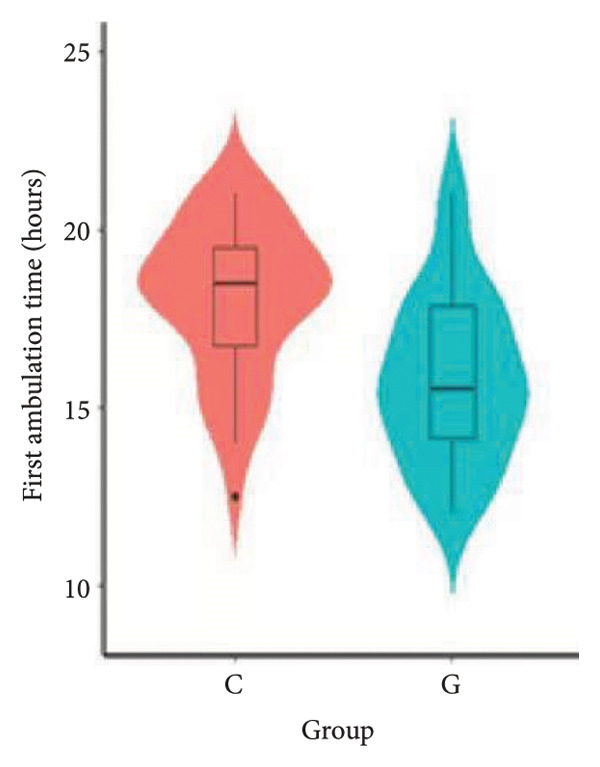
(b)

**Figure:**
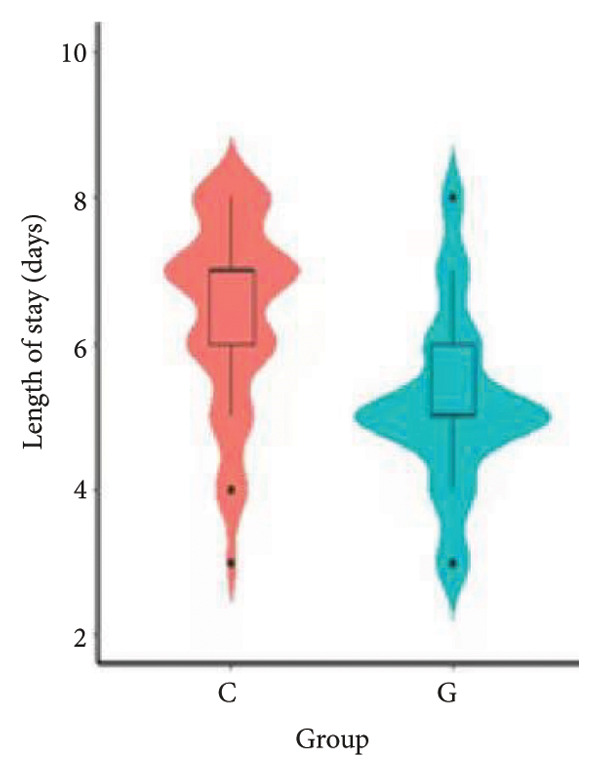
(c)

**Table 2:** Postpartum postoperative rehabilitation index and neonatal outcome.

	Group C (*n* = 35)	Group G (*n* = 34)	*p* value
Adverse reaction (dizziness, nausea, vomiting, chest tightness) (%)	5 (14.3%)	1 (2.9%)	0.20
APGAR 1 min	10.00 (10–10)	10.00 (10–10)	0.32
APGAR 5 min	10.00 (10–10)	10.00 (10–10)	0.32
NICU transfer rate	11 (31.4%)	8 (23.5%)	0.59

Figure 4Comparison of neonatal umbilical artery blood gas between two groups. ns: no statistical significance (*p* > 0.05).

**Figure:**
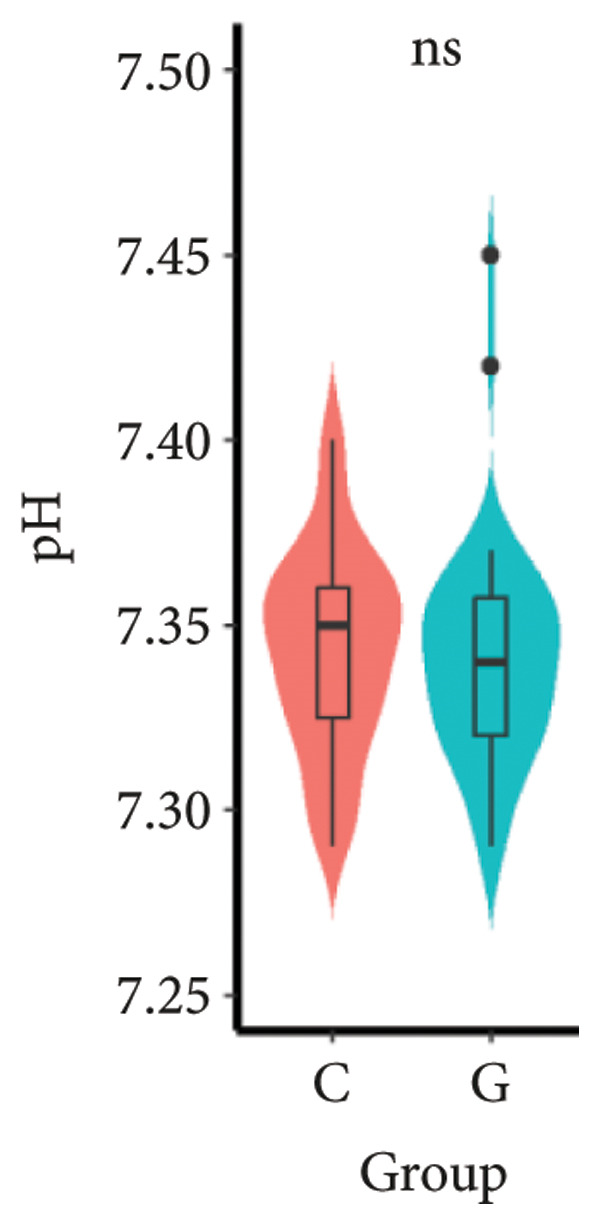
(a)

**Figure:**
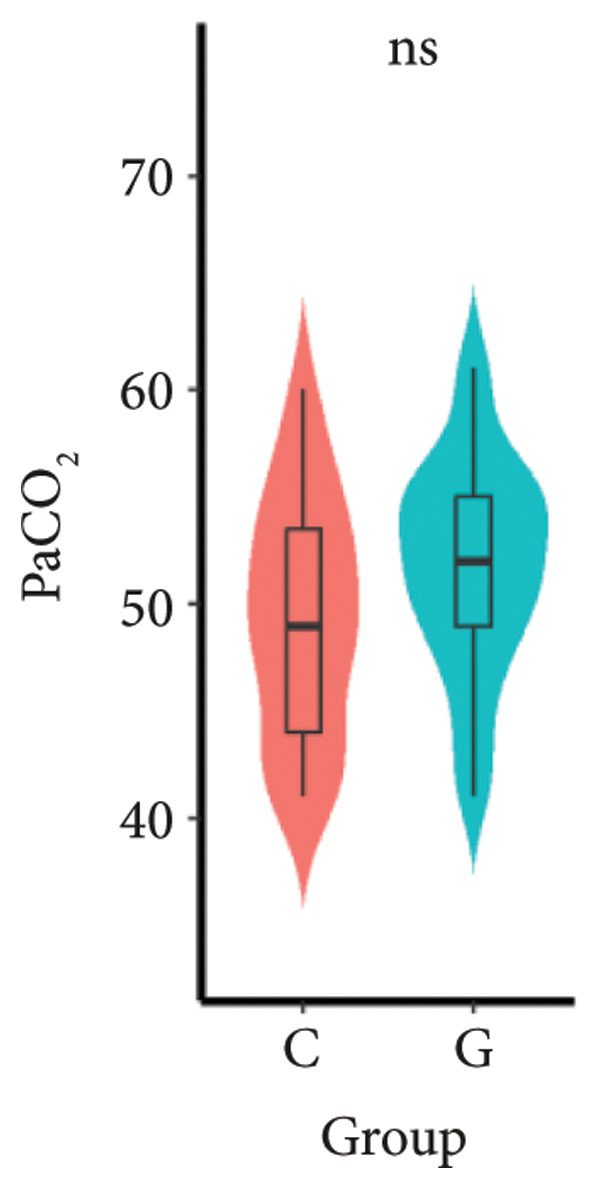
(b)

**Figure:**
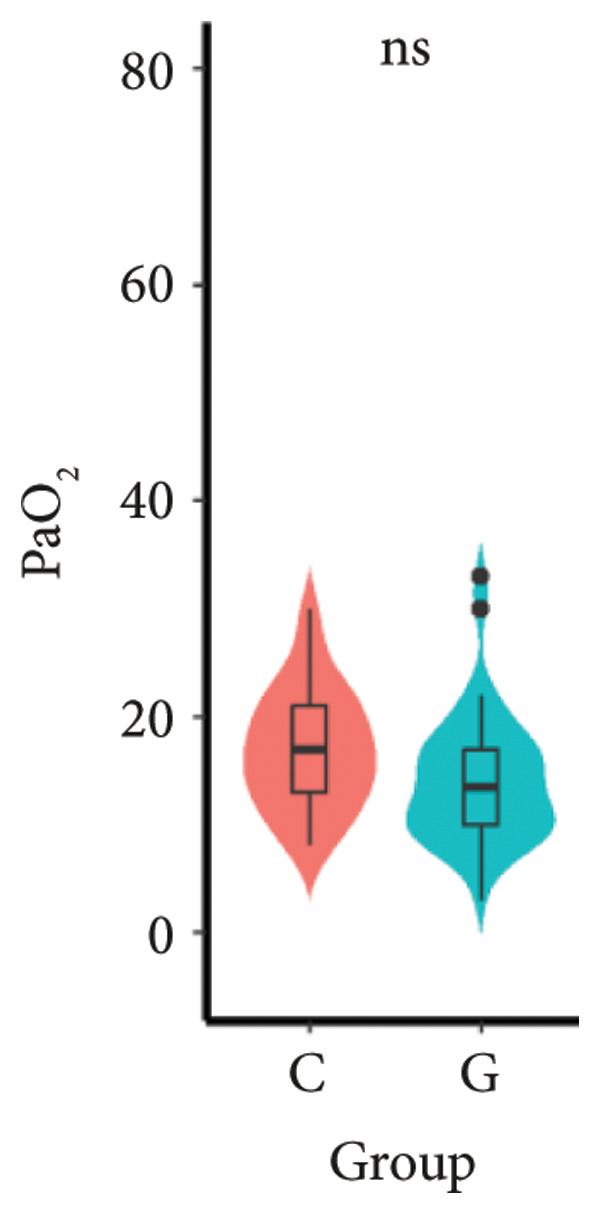
(c)

**Figure:**
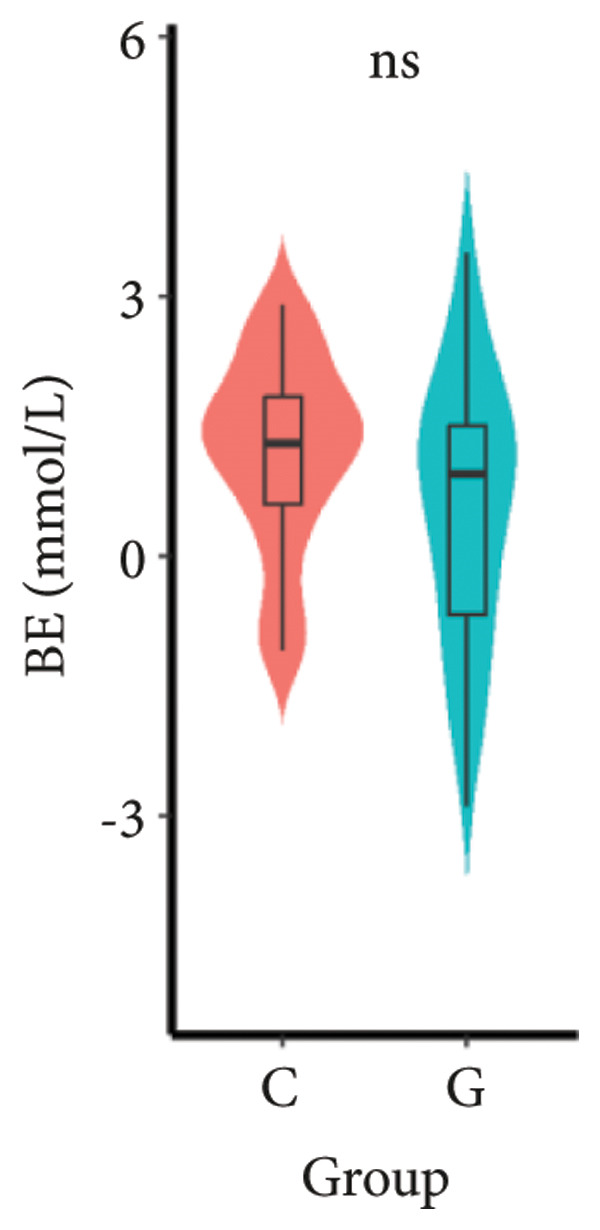
(d)

**Figure:**
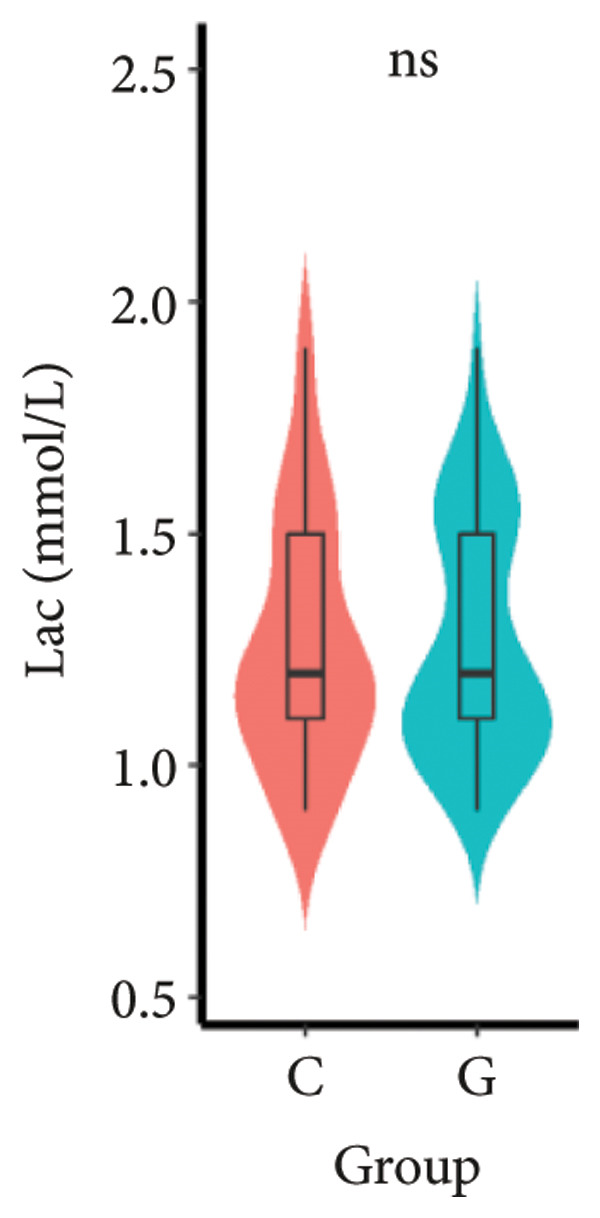
(e)

As a refined fluid management strategy, the core of GDFT is to monitor hemodynamic indicators to guide the amount and speed of infusion, aiming to maintain circulatory stability and prevent the occurrence of hypotension. GDFT is a process of optimizing perioperative SV for individualized fluid therapy by monitoring CO with CO monitor or ultrasound. In recent years, TTE has been applied gradually in the perioperative period, which can be used as a measurement tool of hemodynamic indicators during pregnancy [13, 14]. Best vital to predict the volume status of parturients (reflecting preload changes) is to monitor the variation of SV. If SV increases by more than 10%, the patient has volume reactivity (in the steep part of the Frank–Starling curve), and further volume supplementation is needed. Once the patient had sufficient blood volume (at the Frank–Starling curve plateau), SV increased by < 10% after fluid infusion, indicating no volume reactivity, and volume loading caused by further fluid replenishment was avoided [20].

Xu S et al. [21] showed in a randomized double‐blind study that no single method could completely prevent hypotension during cesarean section, and traditional fluid load had little effect in preventing hypotension and may increase the risk of volume overload such as pulmonary edema. Previously recommended obstetric first‐line vasopressor drugs, including phenylephrine and ephedrine, also have their own defects. Deoxyepinephrine reflexively causes a slow heart rate, so its application in parturients should be particularly cautious. Meanwhile, its effect of reducing maternal CO on placental blood flow will potentially threaten fetal oxygen supply. Ephedrine may be associated with adverse fetal acid–base outcomes [21, 22]. Compared with preloading, co‐loading may be more effective in preventing hypotension after subarachnoid block [23]. Therefore, GDFT was performed immediately after anesthesia in this study. Chirnoaga et al. [24] showed in a study on urethral tissue perfusion in parturients undergoing liver surgery that maintaining relatively stable hemodynamics may depend on the timing of infusion rather than the amount of infusion. GDFT permeates the preoperative, intraoperative, and perioperative periods, accelerated rehabilitation surgery, and is an essential part of promoting rapid postoperative recovery. Multiple studies have found [25–27] that GDFT can effectively reduce complications after major surgery by 25%–50%, as well as which has been proven to maintain intraoperative hemodynamic stability. GDFT can improve CO, optimize microcirculation, maintain the balance of oxygen supply and demand, and reduce the serum lactic acid of parturients [28]. The unexpected drop in CO at T1 in the GDFT group may be attributed to individual variations in fluid responsiveness, leading to initial under‐resuscitation, or vasodilation after anesthesia reducing venous return. Future studies could optimize GDFT strategies to better match individual needs.

However, there are insufficient data on the effect of GDFT on recovery after cesarean section. The incidence of postanesthesia hypotension and the use of vasoactive drugs are significantly reduced in parturients treated with crystal‐oriented fluids. In this study, compared with Group C, the amount of predelivery fluid and intraoperative infusion in Group G was significantly reduced, and the incidence of intraoperative hypotension was significantly decreased. The incidence of hypotension in parturients with GDFT was significantly lower than that in control parturients, but at the same time, the incidence of hypotension still has controllable decline space, indicating that liquid therapy alone may not be able to completely prevent the occurrence of hypotension after subarachnoid block, which requires the combination of prophylactic use of vasoactive drugs and other methods, and further research is needed. In our study, compared with T0, SBP was significantly decreased at T1–T3 in both groups, SV was significantly decreased at T1 in both groups. Compared with Group C, CO was significantly decreased at T1 in Group G. Given the lack of significant differences in neonatal or most maternal outcomes, the clinical significance of reduced hypotension alone suggests that GDFT should be combined with other measures to comprehensively optimize maternal and infant outcomes. The first postoperative flatus time was (36.71 ± 10.65) vs. (31.62 ± 9.19) hours, the first ambulation time was (18.06 ± 2.17) vs. (15.84 ± 2.37), and length of stay was (6.37 ± 1.33) vs. (5.21 ± 1.23) of Group C and Group G, which was not statistically different, but the first postoperative flatus time of the women in Group G was shortened, which has certain clinical significance. According to the ACOG Committee Opinion on umbilical cord blood gas and acid–base analysis, umbilical artery blood gas (acid–base balance indexes) in our trial is within normal range [29, 30]. There were no significant differences in neonatal 1‐min and 5‐min Apgar score, umbilical artery blood gas (other acid–base balance indexes), and NICU transfer rate between the two groups after delivery. Considering that BE is a metabolic indicator, GDFT therapy may improve the metabolic internal environment of the fetus, but there were no significant differences in BE value in our study. Multicenter large‐sample studies are essential to evaluate potential neonatal benefits. Although the shorter time to first flatus in the GDFT group was not statistically significant, it suggests that optimized fluid therapy may promote earlier bowel recovery by reducing bowel edema, consistent with existing ERAS literature. Future studies could further explore the mechanisms.

In previous work, we explored the correlation and consistency of noninvasive pleth variability index (PVI) combined with ultrasound measurement of inferior vena cava collapsibility index (IVC‐CI) in parturients with twin pregnancies undergoing cesarean section, and we have found that ultrasound is valuable for monitoring volume in high‐risk parturients [31, 32]. This study is a further extension. The continuous research findings demonstrate that through personalized fluid therapy strategies, it is possible to ensure that parturients have sufficient circulating blood volume, thereby reducing the risk of hypotension.

This study has the following limitations. Several limitations of this study may have influenced the results and conclusions. Firstly, the small sample size may have led to insufficient statistical power, affecting the generalizability and extrapolation of the findings. Secondly, the short postoperative follow‐up period may have missed some long‐term complications. Future studies should involve larger sample sizes, longer follow‐up periods, and multicenter collaborations to further validate the findings of this study.

In conclusion, SV‐oriented GDFT based on TTE can reduce the incidence of hypotension after subarachnoid block during cesarean section in AMA parturients. GDFT can guide perioperative volume management of AMA parturients in order to optimize maternal and infant outcomes after cesarean section.

## Ethics Statement

This study was examined and approved by the Medical Ethics Committee of Women’s Hospital of Nanjing Medical University with approval (No. 2022KY‐128‐01). All subject participants provided written consent. All methods were carried out in accordance with the Declaration of Helsinki.

## Consent

Please see the Ethics Statement.

## Conflicts of Interest

The authors declare no conflicts of interest.

## Author Contributions

Jun Ni contributed to the central idea and wrote the initial draft of the paper. Huiying Zhang contributed to refining the ideas and finalizing this paper. Chenyang Xu analyzed most of the data. Xiali Qian, Huiling Yu, and Zijun Tian conducted clinical data collection and follow‐up. Shanwu Feng and Mao Mao carried out additional analyses and finalized this paper. Jun Ni and Huiying Zhang contributed equally to this work.

## Funding

This research was independently conducted by the authors without any financial support from external research projects, funding agencies, or organizations in the public, commercial, or nonprofit sectors.

## Supporting Information

Additional supporting information can be found online in the Supporting Information section.

## Supporting information


**Supporting Information 1** CONSORT 2010 Flow Diagram: A CONSORT 2010–compliant flow diagram is provided to systematically illustrate participant progression through the randomized controlled trial (RCT).

**Supporting Information 2** Raw data: The minimal dataset supporting the conclusions of this article is available in accordance with journal/institutional policies.

## Data Availability

All raw datasets generated or analyzed during this study have been deposited as supporting files and are fully accessible for verification alongside this published manuscript.
