# Supplementary material for: Effect of Goal‐Directed Fluid Therapy on Hypotension From Spinal Anesthesia in Older Parturients Having Cesarean Section: A Randomized Controlled Trial
Source: Anesthesiol Res Pract. 2025 Oct 28;2025:2753707. doi: 10.1155/anrp/2753707 (PMC12568773; doi:10.1155/anrp/2753707)
Supplement: Supplementary file 1 — Supporting Information 1 CONSORT 2010 Flow Diagram: A CONSORT 2010–compliant flow diagram is provided to systematically illustrate participant progression through the randomized controlled trial (RCT). [file ANRP-2025-2753707-s001.doc]

**
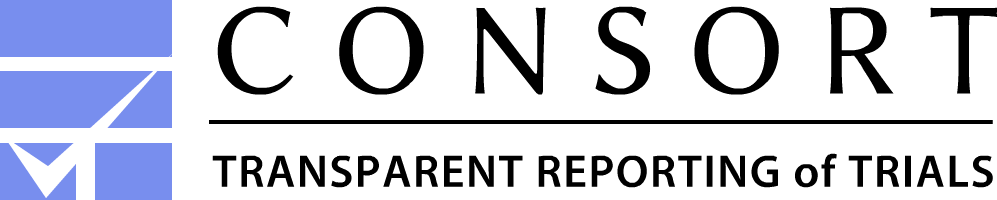
**

**CONSORT 2010 Flow Diagram**

**Allocation**

**Yes**

Assessed for eligibility (n=72)

Drop-out (n=3)

♦  Lost to follow-up(n=2)

♦ Intraoperative massive hemorrhage(n=1)

Group C (n=35)

♦ Before delivery (20 ml ·kg-1 ·h-1 LR )

♦ After delivery (5 ml ·kg-1 ·h-1 LR)

ΔSV＞10%

Group G (n=34)

♦ Challenge test with 3ml ·kg-1 ·3min-1 LR

ΔSV≤ 10%

Randomized (n=69)

5 ml ·kg-1 ·h-1 LR
